# Evaluation of the Extent of Primary Buccal Mucosal Graft Contracture in Augmentation Urethroplasty for Stricture Urethra: A Prospective Observational Study at a Tertiary Healthcare Centre

**DOI:** 10.1155/2021/9913452

**Published:** 2021-07-23

**Authors:** A. BhalaguruIyyan, P. Puvai Murugan, Chandranaath C. Alakhananda, B. M. Zeeshan Hameed

**Affiliations:** ^1^Department of Urology, PSG Institute of Medical Sciences and Research, Coimbatore, Tamil Nadu, India; ^2^Department of Surgery, PSG Institute of Medical Sciences and Research, Coimbatore, Tamil Nadu, India; ^3^Department of Urology, Kasturba Medical College Manipal, Manipal Academy of Higher Education, Manipal, Karnataka, India

## Abstract

**Introduction:**

Buccal mucosal graft (BMG) urethroplasty is considered as gold standard in the treatment of urethral stricture disease. The successful outcome after BMG urethroplasty varies between 66 and 99%. One of the possible causes for failure is BMG contracture. Primary BMG contracture rate is poorly understood and unreported. The present study aimed to evaluate the extent of contracture of buccal mucosa immediately after harvesting.

**Materials and Methods:**

This was a prospective observational study conducted in the Department of Urology at our institute between January 2016 and December 2019. All patients with urethral stricture disease undergoing BMG urethroplasty for the first time were enrolled in the study after obtaining informed consent. Demographic and patient clinical profile was noted. Based on the intraoperative urethral stricture size, the preharvest graft was marked on the buccal mucosa and the size was calculated. Postharvest unstretched size of the graft was measured immediately after graft removal from the oral cavity. Alteration in BMG size was analysed using paired *t*-test.

**Results:**

Forty-four patients were included in the study. Mean age of the patient was 53.6 years. Mean stricture length was 7.45 cm (range 4–12 cm). Mean pre- and postharvest BMG size was 8.3 × 1.5 cm and 7.6 × 1.3 cm, respectively. There was a 8.4% decrease in length and 9.5% decrease in width of the buccal mucosal graft.

**Conclusion:**

Primary buccal mucosal graft contracture is around 8.4% in length and 9.5% in width. It would be better to mark wider than necessary while harvesting buccal mucosa so that tension-free anastomosis is performed.

## 1. Introduction

Buccal mucosal graft (BMG) urethroplasty is considered as the treatment of choice for long segment anterior urethral stricture disease [1]. Easy availability, exposure to moisture, and quick regeneration in the donor area make buccal mucosa a near ideal substitute for the urethra [2]. Still, successful outcomes after BMG urethroplasty are highly variable and range between 66 and 99% [3–6].

The failure of BMG urethroplasty has been attributed to various reasons such as progression of primary disease and loss of vascularity due to over mobilisation of urethra, recurrent infections, and possible contracture of graft.

Graft contracture can be primary or secondary. Primary graft contracture happens immediately after harvest from the donor site is a well-described phenomenon in skin grafts [7]. It can be overcome by increasing the area of the graft from the donor site. However, there is lack of knowledge regarding the extent of primary BMG shrinkage. Hence, in this study, we explored objectively the extent of primary contracture of BMG and its clinical implications in this procedure.

## 2. Materials and Methods

A prospective observational study was conducted in the Department of Urology at a tertiary institute from January 2016 to December 2019 after obtaining the institute's ethical committee approval (project no. 20/128). Patients planned for augmentation urethroplasty using BMG were included in the study, after obtaining consent. Patients' demographic and clinical findings were noted. All cases were operated by a team of three urologists alternating between the buccal graft harvesting and the urethroplasty (BIA, PMP, and ACC). Stricture characteristics were recorded. A thorough oral cavity examination was performed. Patients who had undergone previous oral surgeries or radiation were excluded from the study.

### 2.1. The Procedure-Harvesting of Buccal Mucosal Graft

BMG urethroplasty was performed under general anaesthesia with nasal intubation. After intubation, the patient was in lithotomy position, preliminary urethro-cystoscopy was done using 6/7.5 Fr semirigid ureteroscope, and findings were noted. Through a midline perineal incision, the urethra was identified, isolated, and stricture segment was laid open. The stricture length was measured using a measuring ruler.

**Figure 1:**
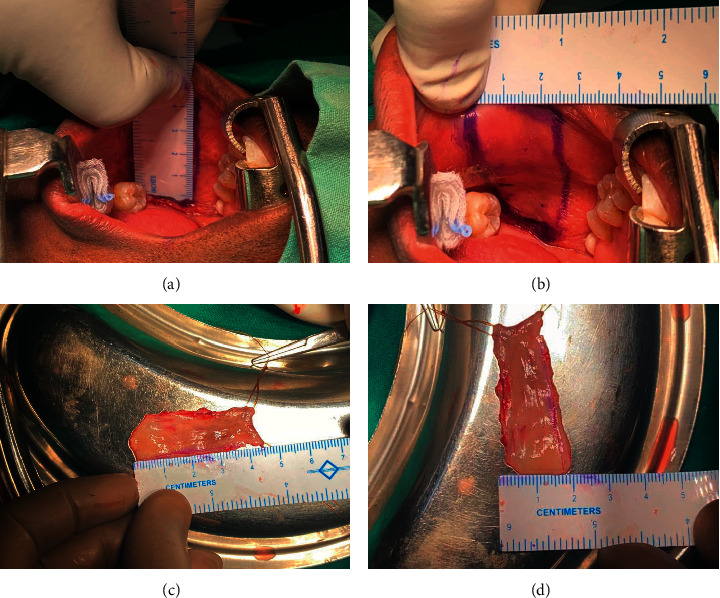
The marking and measurement of the preharvest buccal mucosal graft (a, b) and postharvest measurements of length and breadth (c, d).

BMG harvesting was done in rose position with a sandbag under the shoulder blade and neck extended. Dingman retractor was used to retract the jaws apart. The graft that needs to be harvested was outlined with a marker pen for one centimeter more than the measurement of stricture (Figures 1(a) and 1(b)), taking care to exclude the opening of Stensen's duct. 10 ml solution of 2% lignocaine diluted with adrenaline (1 : 80,000) was injected to achieve hydrodissection. Using a scalpel, the margins of the graft were defined over the pen markings. Holding sutures were taken at the ends of the graft, for better grip. Metzenbaum curved scissor was used to dissect buccal mucosa from underlying buccinator muscle, and the graft was harvested. The donor site haemostasis was secured, was packed with adrenaline-saline soaked gauze pack, and left to heal with secondary intention. The harvested graft was then placed in a kidney tray, and the unstretched length and width of the grafts were measured (Figures 1(c) and 1(d)). The measurement was double-checked by different members of the surgical team to minimise measurement errors. Defattening of the graft was done. The oral gauze pack was removed after 4 hours, and the patient was advised to take fluid diet for 48 hours followed by bland semisolids till they could tolerate normal diet. The oral buccal mucosal wound was inspected every 12 hours for bleeding, wound infection, or restriction of mouth opening till the hospital stay.

### 2.2. Statistical Analysis

Pre- and postharvest size of BMG was compared using a paired *t*-test. Descriptive data were presented in the form of mean, range, percentage, and standard deviation. Statistical analysis was completed using SPSS software, version 21.0 (IBM Corp, NY, USA). Statistical significance was kept below 0.05. The confidence interval was set at 95%.

## 3. Results

Forty four patients who fulfilled the inclusion and exclusion criteria were included in this study. Demographic data are presented in Table 1.

Forty patients (91%) underwent dorsolateral onlay (Kulkarni technique) [8], and 4 (9%) underwent ventral onlay BMG urethroplasty. The mucosal graft was taken from one cheek in 59%, whereas from both cheeks in 41%. The mean operative time for unilateral buccal mucosal graft harvesting was 23 minutes, and for bilateral harvesting, it was 41 minutes.  Table 2 lists the pre- and postharvest measurement of the graft.

**Figure 2:**
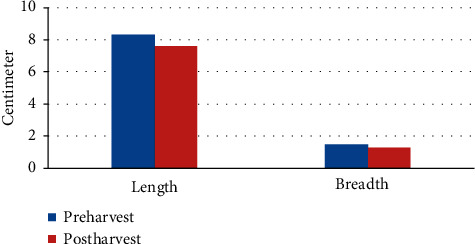
Preharvest vs. postharvest measurements.

Mean preharvest and postharvest BMG length was 8.3 and 7.6 cm, respectively (Figure 2) which showed a statistically significant decrease (8.4%, *p* < 0.001) in length. Mean pre- and postharvest BMG width was 1.5 and 1.3 cm, respectively (Figure 2) which also showed a statistically significant decrease (9.5%, *p* < 0.001) in width.

In patients aged more than 55 years, the mean pre- and postharvest BMG dimensions were 8.9 × 1.5 and 8.2 × 1.3 cm, respectively. While in patients aged less than 55 years, the mean pre- and postharvest BMG dimensions were 8.1 × 1.5 and 7.5 × 1.3 cm, respectively. However, the differences in the contracture between the age groups were not statistically significant (Table 3). Tobacco usage was found in 59% of patients. The mean pre- and postharvest BMG dimensions were 8.3 × 1.5and 7.6 × 1.3 cm, respectively in tobacco users. The mean pre- and postharvest BMG dimensions were 8.4 × 1.4 and 7.7 × 1.3 cm in those not using tobacco. However, the differences in the contracture between both the groups were statistically insignificant (Table 3).

The complications in the donor site are listed in Table 4.

## 4. Discussion

Outcome-related variables in augmentation urethroplasty can be listed as surgical technique, graft quality, stricture characteristics, and patient-related factors. Nontransection of urethra during urethroplasty had better results than transecting urethroplasty [9]. Kulkarni et al. [8] reported that dorsolateral mobilisation of the urethra and preservation of unilateral urethral vascularity is associated with better outcomes of augmentation urethroplasty. Breyer et al. [10] reported that length, site of stricture, and previous surgeries are independent predictors of stricture in multivariate analysis.

Type of graft and graft contracture are other outcome-related variables in augmentation urethroplasty. Skin, bladder, and intestinal and oral mucosa (buccal, lingual, and labial) grafts are the preferred sites of donors for the reconstruction of the urethra [11]. The properties of buccal mucosa such as the absence of hair follicle, highly vascular lamina propria promoting early in growth, wet environment compatibility [12], and ease of harvest with a concealed donor site scar makes it a near ideal substitute for urethra.

The incidence of graft contracture in BMG urethroplasty varies from 3 to 22.5% in various studies [13–18]. Oral mucosal grafts are also used in the ocular reconstructive surgeries [19]. Graft contracture has been noticed in these ocular reconstructive surgeries [19]. It has been reported that graft contracture is less when harvested from hard palate [19].

Graft contracture leads to suboptimal results in BMG urethroplasty. Primary graft contracture, if not accounted for, can lead to overstretching of the graft. This graft overstretching can widen the urethral lumen initially, but in long term, the graft can shrink rapidly, especially after the removal of the catheter. Prior knowledge of the extent of primary graft contracture can give an estimate of extra size of the graft needed. To some extent, primary graft contracture can be dealt by meshing, spreading, and suture fixation of the graft. However, this can be challenging if the size of the urethral defect is large. Surprisingly, no study has focused on this issue in BMG urethroplasty. In our study, we found 8.4% decrease in length and 9.5% decrease in the width of the BMG. As we took one centimeter extrasize graft, tension-free anastomosis was possible. Loss of elastic recoil is the reason for primary graft contracture [20]. The extent of primary skin graft contracture ranges from 9 to 22% [20, 21]. Age is postulated to affect the extent of primary graft contracture in skin grafts [21]. But in our study, age did not influence primary graft contracture of BMG significantly. Smoking and oral tobacco usage is known to cause various intraoral pathologies such as smoker's palate, smoker's melanosis, oral submucosal fibrosis, leukoplakia, leukoedema, and oral cancers [22]. However, the extent of BMG contracture did not vary significantly between tobacco users and nontobacco users. Patel et al. reported that there was not much long-term difference in the restriction of mouth opening after BMG harvest in smokers and nonsmokers [23].

Larger size graft creates larger raw area in the donor site. It leads to inability to close the donor site. Muruganandham et al. [24] reported no changes in long-term graft site morbidity when the graft site is left open or closed. Wood et al. [25] reported no significant difference in obtaining a repeat graft from the buccal mucosal donor site, whether it was closed or left open in the previous harvest. In fact, the severity of pain in the immediate postoperative period is less when the graft site is left open. Immediate postoperative pain (86.4%) is the commonest complication following BMG harvest in our study. Akyüz et al. [26] reported the similar incidence of facial pain (85.7%), while Muruganandam et al. [24] reported to be 83%. The pain and swelling resolves with short duration anti-inflammatory therapy.

Previous studies have reported oral cavity bleeding after BMG harvest in 5–21% [2, 24, 27]. The incidence of persistent oral cavity bleeding is 4.5% in our study. Patel et al. [23] showed that, after achieving complete haemostasis, the buccal mucosal graft site could be left open without suturing to heal by secondary intention and without any significant complications.

Persistent oral numbness (9.1%) and restricted mouth opening (2.3%) are the common long-term sequelae following BMG harvest in our study. Behura et al. [22] in their study noted that the restriction of mouth opening was transient and resolved with time. Castagnetti et al. [27] reported that these complications are not related to the size of graft but are a consequence of dissection of buccinator muscle. They further reported that these complications can be minimised by infiltrating diluted local anaesthesia or saline, aiding hydrodissection of the graft followed by surgical dissection.

Salivatory problems such as excessive salivation or decreased salivation have been reported following BMG harvest [24, 27]. These problems are attributed to parotid/Stensen's duct opening and minor salivary glands injury and are usually transient [27]. None of our patients reported any long-term salivatory problems. Moreover, our patients could tolerate their regular diet within 72 hours, when compared to prior studies [27].

The relatively small sample size was one of the limitations of this study. We did not measure the thickness of the graft and hence could not relate the bearing of graft thickness with the shrinkage rate. Since all the donor sites were left open for healing, we could not compare the morbidity with the donor sites which could have been sutured.

## 5. Conclusion

Primary graft contracture in buccal mucosa is around 8.4% in length and 9.5% in width. It would be better to mark wider than necessary while harvesting buccal mucosa to obtain a tension-free and successful BMG urethroplasty. Donor site morbidity is reasonable if it is left open and allowed to heal by secondary intention.

## Figures and Tables

**Table 1:** Demographic data.

Parameters	Total (*N* = 44)
Age (years)	53.6 (28–74)^*∗*^
Length of the stricture (cm)	7.45 (3–12)^*∗*^
Tobacco users, *n* (%)	26 (59.09)

^*∗*^Data expressed as mean and range in brackets.

**Table 2:** Pre- and postharvest measurement of the graft.

Buccal mucosal graft	Preharvest	Postharvest	Shrinkage in %
Length in cm (mean (±SD))	8.33 (±2.41)	7.64 (±2.34)	8.43 (±2.56)
Breadth in cm (mean (±SD))	1.46 (±0.13)	1.32 (±0.11)	9.51 (±3.69)

**Table 3:** Comparison of factors associated with the contracture of buccal mucosal graft.

Factors	% contracture in length	% contracture in width
Age <55 years (*n* = 32)	8.54	10.10
Age >55 years (*n* = 12)	8.27	8.94
*p* value	0.63	0.38
Smokers/tobacco users (*n* = 26)	8.52	9.60
Nonsmokers/nontobacco users (*n* = 18)	8.24	9.11
*p* value	0.63	0.66

**Table 4:** Postoperative donor site complication.

Donor site complications	Total (*n* = 44)
Immediate complications *n* (%)	
Oral cavity bleeding	2 (4.54)
Pain	38 (86.37)
Facial swelling	28 (63.64)
Restriction of mouth opening	30 (68.18)
Oral numbness	25 (56.82)

Persistent (>4 weeks) complications *n* (%)	
Restriction of mouth opening	1 (2.27)
Oral numbness	4 (9.09)

## Data Availability

The data used to support the findings of this study may be released upon application to the Institutional Human Ethics Committee, PSG Institute of Medical Sciences and Research, post box no. 1674, Peelamedu, Coimbatore 641004, Tamilnadu, India (e-mail: ihec@psgimsr.ac.in).
